# Analysis of Epithelial Growth Factor-Receptor (EGFR) Phosphorylation in Uterine Smooth Muscle Tumors: Correlation to Mucin-1 and Galectin-3 Expression

**DOI:** 10.3390/ijms14034783

**Published:** 2013-02-28

**Authors:** Tobias Weissenbacher, Thomas Vrekoussis, David Roeder, Antonis Makrigiannakis, Doris Mayr, Nina Ditsch, Klaus Friese, Udo Jeschke, Darius Dian

**Affiliations:** 1 Department of Obstetrics and Gynaecology, Innenstadt Campus, Ludwig-Maximilians-University, Munich 80337, Germany; E-Mails: tobias.weissenbacher@med.uni-muenchen.de (T.W.); thomas_vrekoussis@yahoo.gr (T.V.); davidroeder@googlemail.com (D.R.); klaus.friese@med.uni-muenchen.de (K.F.); darius.dian@med.uni-muenchen.de (D.D.); 2 Department of Obstetrics and Gynaecology & Laboratory of Human Reproduction, Medical School, University of Crete, Iraklion 71110, Greece; E-Mail: makrigia@med.uoc.gr; 3 Department of Pathology, Ludwig-Maximilians-University, Munich 80337, Germany; E-Mail: doris.mayr@med.uni-muenchen.de; 4 Department of Obstetrics and Gynaecology, Grosshadern Campus, Ludwig-Maximilians-University, Munich 81377, Germany; E-Mails: nina.ditsch@med.uni-muenchen.de (N.D.); klaus.friese@med.uni-muenchen.de (K.F.)

**Keywords:** myometrium, leiomyoma, leiomyosarcoma, phosphorylation, EGFR, tyrosine kinase

## Abstract

Uterine fibroids are the commonest uterine benign tumors. A potential mechanism of malignant transformation from leiomyomas to leiomyosarcomas has been described. Tyrosine phosphorylation is a key mechanism that controls biological functions, such as proliferation and cell differentiation. The aim of the current study was to evaluate the phosphorylation of epithelial growth factor-receptor (EGFR) in normal myometrium, uterine myomas and uterine leiomyosarcomas. Formalin-fixed paraffin-embedded tissue samples from normal myometrium, leiomyomas and leiomyosarcomas were studied. Samples were immunohistochemically (IHC) assessed using the anti-EGFR phosphorylation of Y845 (pEGFR-Y845) and anti-pEGFR-Y1173 phosphorylation-specific antibodies. IHC staining was evaluated using a semiquantitative score. The expression of pEGFR-Y845 was significantly upregulated in leiomyosarcomas (*p <* 0.001) compared to leiomyomas and normal myometrium. In contrast, pEGFR-Y1173 did not differ significantly between the three groups of the study. Correlation analysis revealed an overall positive correlation between pEGFR Y845 and mucin 1 (MUC1). Further subgroup analysis within the tumoral group (myomas and leiomyosarcomas) revealed an additional negative correlation between pEGFR Y845 and galectin-3 (gal-3) staining. On the contrary no significant correlation was noted within the non-tumoral group. An upregulated EGFR phosphorylation of Y845 in leiomyosarcomas compared to leiomyomas implicates EGFR activation at this special receptor site. Due to these pEGFR-Y845 variations, it can be postulated that MUC1 interacts with it, whereas gal-3 seems to be cleaved from Y845 phosphorylated EGFR. Further research on this field could focus on differences in EGFR pathways as a potentially advantageous diagnostic tool for investigation of benign and malignant signal transduction processes.

## 1. Introduction

Uterine leiomyomas are common pelvic tumors of women of reproductive age [1,2]. Differences occur by race and age. Afro American women have a greater burden regarding fibroids [3–5] compared to Caucasian women. Previous studies have focused on leiomyosarcomas and their origination from either pre-existing leiomyomas or their *de novo* development [6]. Malignant transformation of leiomyomas into leiomyosarcomas seems to be rare, however still debated in literature [7,8]. In addition, Mittal *et al.* demonstrated that leiomyosarcomas can arise from leiomyoma-like areas [9]. Histopathological differentiation between leiomyoma, myoma with pseudosarcomatous features and leiomyosarcoma can be exceedingly difficult [10]. The biology of the development of these mesenchymal malignant tumors is not well understood.

Epidermal growth factor receptor (EGFR) is a receptor tyrosine kinase, a member of the ErbB-family and a regulator of various cellular processes, including cell survival, differentiation, migration and cell growth [11]. The EGFR is implicated in pathological processes, such as oncogenesis, and is linked with a poor prognosis in several epithelial carcinomas [12]. Inhibition of uncontrolled EGFR expression improved treatment of malignant diseases, such as breast and lung cancers [13]. The investigation of EGFR and its signaling pathway is, therefore, important in research concerning the tumor biology of this entity.

There is evidence that mucin-1 (MUC1) has a regulatory function in the trafficking and nuclear activity of EGFR [12]. Recently, we demonstrated that epithelial mucin-1 (MUC1) was upregulated in leiomyomas and leiomyosarcomas compared to normal myometrium [14]. Interestingly, it was also shown that MUC-1 and EGFR may be regulated by galectin-3 in pancreatic carcinoma [15]. To this direction, Gal-3 has also been reported to interact with MUC1 and EGFR, acting as a bridge between MUC1 and EGFR [16]. Since EGFR and MUC1 are now considered ligands for Gal-3, it could be hypothesized that these three molecules could form a regulatory network, necessitating the study of this molecule in parallel, rather than separately.

EGFR phosphorylation is regulated by dephosphorylation and transphosphorylation of receptors by tyrosine phosphatases.

Phosphorylation of EGFR on tyrosine 845 is accompanied with activation of this receptor tyrosine kinase. It is known to be responsible for oncogenetic processes [11] and is required for the transactivation of EGFR [17,18]. Furthermore, phenylalanine substitution of Y845 (Y845F) was found to inhibit EGF-induced DNA synthesis, making Y845 a potential target in oncological treatment decisions [11].

The Y1173 EGFR phosphorylation was investigated recently. An interaction of this phosphorylation site with the EGFR has been described, supporting it as a promising therapeutical target in breast and lung cancer [13].

The role of EGFR phosphorylation has not been investigated so far in leiomyomas and leiomyosarcomas. Since galectins have been reported as being involved in tumor development, investigation of their interaction with phosphorylated EGFR (pEGFR)-Y845 and -Y1173 may also be important.

The aim of this study was to evaluate differences in the EGFR activation by phosphorylation in myomas and leiomyosarcomas. We additionally aimed to analyze potential correlations between the EGFR phosphorylations under study and the expressions of MUC1 and Gal-3.

## 2. Results and Discussion

### 2.1. EGFR-Y845

**Figure 1:**
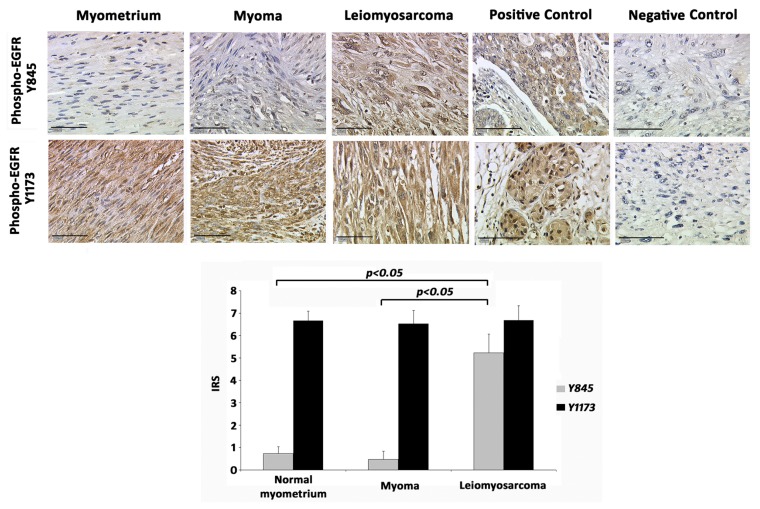
Representative microphotographs of the molecules currently studied in myometrium, myoma and leiomyosarcoma cases. As shown, both in the microphotographs and in the graph, the epithelial growth factor-receptor (EGFR) phosphorylation of Y845 is significantly more abundant in leiomyosarcomas compared to myomas and normal endometrium (*p* < 0.05). Positive control (breast cancer) and negative control (leiomyosarcoma stained with IgG isotype control serum). Bar = 100 μm.

In normal myometrium, pEGFR-Y845 staining was either absent or weak (mean International Remmele Score (IRS) = 0.73 ± 0.30). A total of 17 patients with myomas were investigated for pEGFR-Y845 staining. Only two cases (11.7%) were positive for the phosphorylated EGFR in this position, yielding a mean IRS = 0.47 ± 0.36 (Figure 1). In contrast, all cases with leiomyosarcomas showed a strong staining, with a mean IRS = 5.22 ± 0.84 (Figure 1), differing highly significantly both from myometrium (*p* < 0.001) and myoma (*p* < 0.001) pEGFR-Y845 expression.

### 2.2. EGFR-Y1173

In normal myometrium, pEGFR-Y1173 staining was found to be moderate (mean IRS = 6.66 ± 0.42). A total of 21 patients with myomas were investigated for pEGFR-Y1173 staining. In all cases, we identified a medium to strong reactivity for pEGFR-Y1173 in myomas (mean IRS = 6.52 ± 0.58) (Figure 1). All cases of leiomyosarcomas also showed a comparable pEGFR-Y1173 reactivity (mean IRS = 6.66 ± 0.66) (Figure 1). None of the groups differed significantly from the others in terms of pEGFR-Y1173 expression.

### 2.3. Correlation Analysis

In order to perform the so described correlation analysis, the corresponding data for the patients participating in the current study (being a part of a previous study published by our group [14] regarding MUC1 and Gal-3 expression, were retrieved. Initial correlation analysis by merging the tumoral (myoma and leiomyosarcoma) and the non-tumoral (myometrium) cases revealed a positive correlation between the EGFR phosphorylation of Y845 (pEGFR-Y845) and MUC1 (Spearman r, *p* = 0.009). Further subgroup analysis within the tumoral cases revealed that the EGFR Y845 (pEGFR-Y845) correlated positively with the MUC1 expression (Spearman r, *p* = 0.003). A significant negative correlation could be also demonstrated between pEGFR-Y845 phosphorylation and Gal-3 expression (Spearman r, *p* = 0.010). Correlation analysis did not reveal any significant correlation within the non-tumoral group.

The activation of EGFR by the phosphorylation of Y1173 (pEGFR-Y1173) did not correlate significantly neither with pEGFR-Y845 (*p* = 0.827) nor with MUC1 (*p* = 0.195)/Gal-3 (*p* = 0.15) stainings.

### 2.4. Discussion

The role of EGFR along with vascular endothelial growth factor (VEGF) were already investigated in leiomyomas and leiomyosarcomas [19]. It was concluded that angiogenic growth factors seem to play an important role in the induction of malignant transformation of leiomyosarcomas. The overexpression of EGFR in uterine leiomyosarcomas compared to uterine myomas was also demonstrated in mice [20]. Sato *et al.* demonstrated an EGFR overexpression in human soft tissue sarcomas. A total of 73% of leiomyosarcomas had a positive EGFR staining, whereas lower expression was described in liposarcomas [21]. Recently, the activation of EGFR pathways—as this was shown by pEGFR-Y1173 staining—in leiomyosarcoma cases was reported [22]. Treatment of testicular leiomyosarcoma cells with the EGFR inhibitor, gefitinib, combined with vincristine succeeded in reducing tumor size by increasing the tumor cell apoptotic rate [22]. Additionally, an elevated pEGFR-Y1173 activation was shown in leiomyosarcoma cases compared to myometrium cases, which served as controls [22]. Our results are in partial concordance with these findings. We could verify the moderate to strong expression of pEGFR-Y1173 in leiomyosarcoma cases. However, in our small series, pEGFR-Y1173 was found in comparable rates in both myomas/leiomyosarcomas, as well as in normal myometrium. The disagreement regarding the normal myometrium between our results and the results presented by Sette *et al.*[22] highlights the necessity of a large case series, which will clarify the role of the pEGFR-Y1173 in tumorigenesis. After all, both the myometrium samples presented by us and by Sette *et al.* are quite small. Thus, conclusions are to be made with caution.

To the best of our knowledge, this is the first time that pEGFR-Y845 activation was demonstrated in leiomyosarcoma. Interestingly, this upregulation was characteristic only for leiomyosarcomas, since both myomas and normal myometrium presented with weak immunoreactivity. This finding may indicate a role for pEGFR-Y845 in the transformation of both myometrium and myomas to leiomyosarcoma. This, in turn, could support the existing theories of leiomyosarcoma tumorigenesis, claiming that this malignancy may initiate either *de novo* or from an existing myoma. Although we could not show a differential pEGFR-Y1173 immunoreactivity, by combining the results of Sette *et al.*[22] with the ones presented herein, we can hypothesize that EGFR activation by phosphorylation on multiple sites could contribute to leiomyosarcoma tumorigenesis and chemoresistance; thus, such phosphorylation sites could be major candidates for designing efficient interventions with potential clinical impact.

Nonetheless, the current findings are more supportive for pEGFR-Y845 to play a role in EGFR activation, at least in uterine leiomyosarcoma. The phosphorylation of Y845 on EGFR was previously reported to be an important regulator in oncogenetic pathways by binding proteins, such as cytochrome c oxidase II [11]. Additionally, EGFR Y845 phosphorylation has been shown to be in a crosstalk with c-Src, inducing proliferation of breast cancer cells [23]. This interaction between pEGFR-Y845 and c-Src has been reported to be important in proliferation and tumorigenesis [24].

Herein, a positive correlation between pEGFR-Y845 and MUC1 was demonstrated. A regulatory function of MUC1 on EGFR was already described by Bitler *et al.* They reported that the presence of MUC1 results in significant co-localization of EGFR and influences the association of EGFR with transcriptionally active promoter regions [12]. Supporting our results, Li *et al.* reported the co-expression of both MUC1 and EGFR; this further strengthens the herein presented MUC1/pEGFR-Y845 correlation [25]. No correlation was highlighted between Y1173 and any of the rest of the molecules of the current study. Nevertheless, it is an alternative way of EGFR activation being related to oncogenic pathways; activated c-Src phosphorylates the EGFR at Y1173 [26].

Interestingly, in tumoral cases (myomas and leiomyosarcomas), but not in myometrium, pEGFR-Y845 proved to correlate negatively with Gal-3. This significant negative correlation could imply a possible downregulation of Gal-3 by pEGFR-Y845, with an additional promoting result in oncogenesis. This hypothesis could be further supported by the fact that loss of Gal-3 is reported to be associated with the progression and invasive potential of metastatic cells [27]. Reduction in Gal-3 has also been demonstrated in ovarian carcinogenesis, since Gal-3 immunostaining was found to decrease significantly in the transition from cystadenomas and serous borderline tumors to carcinomas [28]. Thus, the negative correlation between pEGFR-Y845 and Gal-3, shown herein in tumoral cases, highlights a potential EGFR regulation of galectin-3, this being possibly important in the transformation of myomas to leiomyosarcomas.

Our findings, although clearly supporting a phospho-EGFR-Y845/MUC1/Gal-3 correlation, should be taken with caution. Larger samples of uterine leiomyosarcomas may be necessary to clarify the extent to which phospho-EGFR-Y845 contributes to uterine leiomyosarcoma transformation. Further studies are also needed in order to assess the role of this phosphorylation in clinical outcomes (disease free and overall survival).

## 3. Experimental Section

### 3.1. Study Population

Tissue samples from normal myometrium, leiomyomas or leiosarcomas were obtained from the archives of the First Department of Obstetrics and Gynecology, Innenstadt Campus, Ludwig-Maximilian-University Munich, Germany. A total of 30 women with uterine myomas (*n* = 21) or leiomyosarcomas (*n* = 9) were included in the study. Another 15 cases of women that underwent hysterectomy due to benign endometrial pathology were included as controls (normal myometrium). The EGFR phosphorylation of Y845 (pEGFR-Y845) was investigated in 15 cases of normal myometrium, in 17 cases of myomas and in 9 cases of leiomyosarcomas. The EGFR phosphorylation of Y1173 (pEGFR-Y1173) was investigated in 6 cases of normal myometrium, in 21 cases of myomas and in 9 cases of leiomyosarcomas. The institutional review board of the Ludwig-Maximilian-University Munich, Germany, approved the study, and all the patients gave their informed consent.

### 3.2. Immunohistochemistry

Formalin-fixed paraffin-embedded tissue sections were deparaffinized in xylol for 15 min, rehydrated in a descending series of alcohol (100%, 70% and 50%) and subjected to epitope retrieval for 10 min in a pressure cooker using sodium citrate buffer (pH 6.0) containing 0.1 M citric acid and 0.1 M sodium citrate in distilled water. After cooling, the sections were washed twice in phosphate buffered saline solution (PBS). Non-specific binding of the primary antibodies was blocked by incubating the sections with diluted normal serum (10 mL PBS containing 150 μL horse serum; Vector Laboratories, Burlingame, CA, USA) for 20 min at room temperature. The sections were then incubated at room temperature for 60 min with the primary antibodies. The primary antibodies used along with their features are presented in Table 1. Reactivity was revealed using the appropriate Vectastain Elite ABC kit (Vector Laboratories, Burlingame, CA, USA), according to the manufacturer’s protocol. Visualization was performed using substrate and the chromogen 3,3′-diaminobenzidine (DAB; Dako, Glostrup, Denmark) for 2 min. The sections were then counterstained with Mayer’s acidic hematoxylin and dehydrated in an ascending series of alcohol (70%–100%). After xylol treatment, the sections were covered. Negative controls were performed by replacing the primary antibody with the appropriate IgG isotype control serum. Breast cancer tissue sections were used as positive control.

The intensity and distribution patterns of specific immunohistochemical staining were evaluated using the semiquantitative International Remmele Score (IRS), as described elsewhere, to assess the expression pattern of other molecules, such as steroid receptors, glycodelin and MUC1 [29–31]. The IRS score was calculated by multiplication of the optical staining intensity (graded as 0 = no, 1 = weak, 2 = moderate and 3 = strong staining) and the percentage of positively stained cells (0 = no staining, 1 = <10% of cells, 2 = 11%–50% of cells, 3 = 51%–80% of cells and 4 = >81% of cells stained). The slides were scored by two observers by consensus. Sections were examined using a Leitz (Wetzlar, Germany) microscope equipped with a 3CCD color camera (JVC, Victor Company of Japan, Yokohama, Japan).

### 3.3. Statistics

The results were evaluated using the non-parametric Mann-Whitney-U test. The non-parametric Spearman correlation coefficient was used for estimating correlation between MUC1 and Gal-3 and the phosphorylations of EGFR (pEGFR-Y845 and pEGFR-Y1173) (IBM SPSS, New York, NY, USA). Significance was assumed at *p* < 0.05.

## 4. Conclusions

Therapeutic strategies, such as the use of trastuzumab and other tyrosine kinase inhibitors, have already been approved for targeted treatment of cancer. This targeted therapy is currently limited to certain cancers in clinical practice. The blockade of EGFR as an important therapeutical intervention alone and in combination with chemotherapy has already been investigated in soft tissue sarcomas (including uterine leiomyosarcomas) using *in vitro* and *in vivo* (animal model) experiments with promising results [32]. Our findings further support these results and highlight a role for EGFR and especially for the activation by phosphorylation of Y845, as far as uterine leiomyosarcoma is concerned.

## Figures and Tables

**Table 1:** Salient features of the antibodies used in the current study.

Antibody	Clone	Isotype	Concentration	Source
pEGFR-Y845	polyclonal	Rabbit IgG	1.0 mg/mL	R & D Systems, USA
pEGFR-Y1173	polyclonal	Rabbit IgG	1.0 mg/mL	R & D Systems, USA
Galectin-3	9C4	Mouse IgG	2.0 μg/mL	Novocastra, UK
Mucin-1	VU-4-H5	Mouse IgG1	6.8 μg/mL	Invitrogen, USA
